# Assessing the impact of body composition, metabolic and oxidative stress parameters on insulin resistance as a prognostic marker for reactive hypoglycemia: a cross-sectional study in overweight, obese, and normal weight individuals

**DOI:** 10.3389/fphar.2024.1329802

**Published:** 2024-04-09

**Authors:** Maria Kościuszko, Angelika Buczyńska, Katarzyna Łuka, Ewa Duraj, Katarzyna Żuk-Czerniawska, Agnieszka Adamska, Katarzyna Siewko, Aleksandra Wiatr, Adam Jacek Krętowski, Anna Popławska-Kita

**Affiliations:** ^1^ Department of Endocrinology, Diabetology and Internal Medicine, Medical University of Bialystok, Bialystok, Poland; ^2^ Clinical Research Center, Medical University of Bialystok, Bialystok, Poland; ^3^ Department of Periodontal and Oral Mucosa Diseases, Medical University of Bialystok, Bialystok, Poland

**Keywords:** oxidative stress, insulin resistance, reactive hypoglycemia, obesity, overweight

## Abstract

Oxidative stress (OS) plays a pivotal role in the pathogenesis of insulin resistance (IR), particularly in its association with obesity. This study evaluate both the diagnostic and clinical significance of assessing oxidative status in patients affected by overweight and obesity displaying IR, especially with reactive hypoglycemic episodes (RH). A comprehensive examination of OS biomarkers was carried out, encompassing measurements of total oxidative capacity (TOC) and total antioxidant capacity (TAC). Our analysis results reveal noteworthy connections between OS levels and the severity of IR in overweight and obese patients. Moreover, in the study, we demonstrated the diagnostic utility of serum concentrations of TAC and TOC as indicators of the risk of RH, the occurrence of which, even at the stage of overweight, may be associated with increased OS and further development of obesity. Our findings imply that the evaluation of oxidative status could serve as a crucial diagnostic and prognostic tool for patients observed with IR and overweight and obesity. In conclusion, our study underscores the potential utility of assessing oxidative status in the context of IR and highlights the possibility of identifying novel therapeutic targets for the treatment of overweight and obese patients.

## 1 Introduction

The global obesity crisis represents an urgent and rapidly intensifying public health challenge, affecting a significantly alarming portion of the global population, impacting over two billion individuals worldwide (Stierman et al., 2021). This widespread prevalence of obesity poses a significant threat to public health systems and demands meticulous attention. Obesity, as defined by the World Health Organization (WHO), is characterized by a body mass index (BMI) of 30 or higher and is intricately enmeshed with a spectrum of metabolic disorders (Weir and Jan 2023). It stands as a prominent precursor to an array of health complications, foremost among them being insulin resistance (IR) (Safaei et al., 2021). IR occupies a central and defining role in the pathogenesis of a wide spectrum of metabolic disorders, and its prevalence is notably elevated among individuals contending with excessive body weight. Within the intricate web of obesity and IR, oxidative stress (OS) assumes a substantial and interconnected role of paramount significance. Moreover there is a potential intricate relationship between reactive hypoglycemia (RH) and OS, but their exact mechanisms remain unclear and are the subject of further research. The obese state instigates a complex cascade of physiological events culminating in an elevated state of OS. This elevated OS primarily emanates from the release of pro-inflammatory cytokines and other molecules originating from adipose tissue, consequently fostering the production of reactive oxidant species (ROS) within the biological system (Sies, 2015). In a reciprocal manner, OS can exacerbate IR by disrupting insulin signaling pathways and inciting a state of chronic inflammation within the organism (Yazıcı and Sezer, 2017; Avgerinos et al., 2019; Luc et al., 2019). This intricate interplay between OS and IR underscores the profound importance of investigating and comprehending their symbiotic relationship, as it harbors the key to deciphering the intricate mechanisms that underlie metabolic disturbances in obesity. Assessing oxidative status is a crucial tool for clinicians, offering insights into the health of individuals with obesity. Elevated OS levels are closely tied to higher risks of chronic conditions, including cardiovascular disease, type 2 diabetes mellitus (T2DM), and certain cancers (Tanase et al., 2020; da Silva et al., 2020; Reaven, 2012). Monitoring oxidative status enables tailored interventions. Individuals with obesity and high OS levels may benefit from dietary adjustments, antioxidant supplements, or specific medications, while those with lower OS levels may require different approaches (Sies, 2015). Consistently monitoring oxidative status allows clinicians to gauge treatment effectiveness. Improvements in oxidative status serve as a valuable marker of treatment success, aiding both patients and healthcare providers in tracking progress and making necessary treatment adjustments. Since OS levels can vary among individuals with obesity, a personalized approach that considers an individual’s oxidative status is essential. Furthermore, researching oxidative status in the context of obesity has the potential to yield new diagnostic tools and treatments. A deeper understanding of the molecular mechanisms linking OS, RH and obesity could uncover novel targets for drug development and innovative treatment strategies, ultimately improving the clinical management of this multifaceted condition.

Hence, this study aims to investigate the clinical significance of assessing OS in overweight and obese individuals with IR especially with RH, with the goal of identifying new medical targets for obesity treatment. This research endeavor is poised to provide invaluable insights into the effective management and potential prevention of the array of metabolic disorders intricately linked with obesity.

## 2 Material and methods

### 2.1 Study designe

The study encompassed a comprehensive assessment of various parameters involving 150 participants, meticulously categorized into three distinct groups. The study’s participant cohort was stratified into three groups: comprised 50 overweight participants with IR (defined as BMI >25 kg/m^2^) (O1), the second group consisted of 50 individuals presenting both obesity and IR (defined as BMI >30 kg/m^2^) (O2) and the third group encompassed 50 healthy volunteers with a BMI below 25 kg/m^2^ (CG). Anthropometric measurements, including height and weight, were meticulously conducted employing standardized instruments, ensuring precision and accuracy. Body mass index (BMI) was then calculated by dividing body weight (in kilograms) by the square of height (in square meters). Insulin sensitivity was assessed using fasting insulin and glucose levels for the calculation of the homeostasis model assessment (HOMA), while the HOMA-IR index (fasting insulin level (μU/mL) multiplied by fasting blood glucose level (mmol/L) divided by 22.5) was utilized as a measure of IR. Furthermore, a thorough evaluation of body composition was executed employing the dual-energy X-ray absorptiometry (DXA) method, ensuring precise measurements. The patients did not smoke cigarettes and did not abuse alcohol, did not take any medications including hyperglycemic, hypoglycemic, immunosuppressive drugs, and did not have any other conditions that could have affected peripheral OS or similar criteria. The above information was obtained based on the medical history interview, physical examination and medical documentation provided by the patients. Venous blood (5.5 mL) was obtained and centrifuged, with serum subsequent separation and then frozen at − 80°C.

### 2.2 Biochemical measurements

To assess oxidative status, the study relied on the quantification of total oxidative capacity (TOC) and total antioxidant capacity (total oxidative capacity). Specifically, the TOC status was determined through a photometric immunodiagnostic assay employing the PerOx (TOS/TAC) kit sourced from KC5100 in Bansheim, Germany. IR was assessed using the homeostatic model assessment for IR (HOMA-IR) and further validated through an oral glucose tolerance test (OGTT). During the OGTT, glucose and insulin measurements were obtained at specific intervals (0, 60, 120, 180, and 240 min). The criterion for hypoglycemia was established as a glucose concentration below 70 mg/dL (Ibrahim et al., 2020). The enzymatic colorimetric method on a Roche C111 device (Roche Diagnostics, Basel, Switzerland) was utilized to assay concentrations of glucose. The insulin, C-peptide and 25-OH vitamin D concentrations were measured using the electrochemiluminescence (ECLIA) method on a Roche E411 device (Roche Diagnostics, Sussex, UK). The glycated hemoglobin (HbA1c) was tested on Bio-Rad D10 dual HbA2/F/A1c platform, which uses the CE-HPLC method. To uphold the integrity of the analyses, samples and controls were processed using the blind analysis method within a single run, minimizing the potential for bias.

### 2.3 Bioleletrical impedance anlysis

The Bioelectrical Impedance Analysis (BIA) method was employed to assess body composition using the medical body analyzer INBODY 220 (Biospace, Korea). This device enables the measurement of body mass, total body water (TBW), fat mass, skeletal muscle mass, BMI and resting metabolic rate (RMR).

## 3 Statistical analysis

Statistical analyses were executed using GraphPad Prism 9.0 software. The initial assessment of data distribution, conducted using the Shapiro-Wilk test, revealed that the examined parameters did not conform to a normal distribution. Consequently, nonparametric tests were employed for inter-group comparisons. The Mann-Whitney (**) and Kruskal–Wallis (*) tests for independent samples were applied to utilized to discern statistically significant differences in clinical parameters among the various study groups, with statistical significance defined as *p* < 0.05. Furthermore, Spearman correlation analysis was conducted to assess the relationships between the studied parameters. Finally, receiver operating characteristic (ROC) curves were generated, and the area under the ROC curve (AUC) was analyzed to evaluate the clinical utility of the studied parameters in comparison to an AUC of 0.05. Furthermore, odds ratios (ORs) and logistic regressions were computed using also GraphPad Prism v. 9.0.

## 4 Ethical committee

The procedures were approved by the Local Ethics Committee of the Medical University of Bialystok, Poland, and written informed consent was obtained from each participant (APK.002.364.2021).

## 5 Results

### 5.1 Measurement analysis

The all studied groups exhibit significant differences in terms of BMI, body weight, fat mass, waist-to-hip ratio (WHR), and RMR (*p* < 0.001) (Table 1).

**TABLE 1:** Basic characteristics of the study group in terms of BMI, WHR and RMR.

Parameter	O1	O2	CG	O1 vs. CG**	O2 vs. CG**	O1 vs. O2**
BMI (kg/m^2^)	28 (25.5–29.9)	37 (30.1–52.2)	22 (19.5–25)	**<0.001**	**<0.001**	**0.009**
Body weight (kg)	78 (61.5–107.8)	106.7 (78.9–149)	62.5 (51.5–87.4)	**0.017**	**<0.001**	**<0.001**
Fat mass (kg)	27.1 (19.7–46.6)	33.6 (22.3–52.6)	24.4 (19.7–37.4)	**<0.001**	**<0.001**	**<0.001**
Muscle mass (kg)	26.7 (14.5–41.8)	44.9 (25.6–98.6)	16.5 (8.0–23.1)	**<0.001**	**<0.001**	**<0.001**
TBW	35.8 (27.3–60.1)	44.5 (31.4–67.6)	32.4 (27.0–48.3)	**<0.001**	**<0.001**	**<0.001**
WHR	0.95 (0.83–1.09)	1.03 (0.84–1.3)	0.87 (0.79–0.96)	**<0.001**	**<0.001**	**<0.001**
RMR (kcal)	1,386 (1,112–1947)	1,604 (1,266–2,224)	1,237 (1,126–1,601)	**<0.001**	**<0.001**	**<0.001**

**BMI,** body mass index; **TBW**, total body water; **WHR**, waist to hips ratio; **RMR**, resting metabolic rate, **- Mann Whitney measurements; **O1**; overweight group; **O2**, obese group; **CG**, control group.

### 5.2 Biochemical analysis

In the group of O1 patients, statistically significant higher concentration of HOMA-IR, glucose 60′, 120′ and 180′, insulin 0′, 60′, 180′ and 240′ and C-peptide were observed compering to healthy volunteers, respectively. Moreover, compering O1 to O2 group the significant increased concentration of HOMA-IR, glucose 60′, 120′ and 180′ and insulin 0′, 60′, 120′, 180 ′and 240′ and C-peptide were noticed. Accordingly, in the group of patients O2 the increased concentration of HOMA-IR, glucose 0′, 60′ and 180’, insulin 0′, 60′, 120′, 180 ′and 240′ and C-peptide were observed compering to healthy volunteers, respectively (Table 2).

**TABLE 2:** The characterization of the investigated parameters.

Parameter	O1	O2	CG	O1 vs. CG**	O2 vs. CG**	O1 vs. O2**
HOMA-IR	4.89 (2.69–6.76)	6.56 (3.9–6.43)	1.69 (0.82–2.33)	**<0.001**	**<0.001**	**0.029**
Glucose 0 (mg/dL)	99.7 (84.0–118.0)	105.6 (88.0–124.0)	95.2 (82.0–105.0)	0.213	**0.043**	0.058
Glucose 60 (mg/dL)	127.0 (67.1–283)	163.3 (64.5–285)	123.7 (76.2–214)	**0.046**	**<0.001**	**0.019**
Glucose 120 (mg/dL)	118.2 (50.0–165.0)	140.0 (59.0–196.0)	112.8 (76.0–141.0)	**0.048**	**0.012**	**0.023**
Glucose 180 (mg/dL)	63.9 (49.0–115.0)	69.0 (47.0–189.6)	79.2 (71–117)	**<0.001**	**<0.001**	**0.037**
Glucose 240 (mg/dL)	82.4 (61.0–105.0)	80.0 (60.0–111.0)	81.5 (72.0–100.0)	0.071	0.054	0.062
Insulin 0 (uU/mL)	10.17 (4.1–29.1)	20.0 (5.5–60.2)	7.76 (1.5–15.9)	**0.034**	**<0.001**	**0.019**
Insulin 60 (uU/mL)	89.3 (22.1–215.3)	155.7 (12.4–439.8)	77.39 (18.7–152.1)	**0.035**	**<0.001**	**0.011**
Insulin 120 (uU/mL)	53.2 (4.6–153.0)	98.3 (5.7–472.0)	54.2 (7.2–80.0)	0.057	**<0.001**	**0.015**
Insulin 180 (uU/mL)	11.3 (2.2–43.2)	14.6 (1.2–69.3)	9.1 (3.5–16.0)	**0.041**	**0.038**	**0.046**
Insulin 240 (uU/mL)	6.74 (1.9–37.2)	14.6 (2.1–92.0)	4.9 (1.5–13.6)	**0.047**	**<0.001**	**0.026**
C-peptide (ng/mL)	2.3 (1.4–3.9)	3.4 (1.7–9.2)	1.9 (1.2–3.8)	**0.049**	**<0.001**	**0.031**
HbA1c (%)	5.4 (4.9–6.3)	5.5 (4.9–6.5)	5.4 (4.8–6.1)	0.125	0.543	0.642
25-OH Vitamin D (ng/mL)	31.4 (8.7–82.9)	29.8 (9.2–67.3)	32.3 (15.4–67.1)	0.471	0.515	0.646

**HOMA-IR,** homeostatic model assessment for Insulin Resistance, **HbA1c**, glycated hemoglobin; **- Mann Whitney measurements, **O1**; overweight group; **O2**, obese group; **CG**, control group.

### 5.3 Hypoglicemic episodes

During our study, we observed that in the O1 group, there were four episodes of hypoglycemia within 120 min from the start of the OGTT. Furthermore, within 180 min from the OGTT, a total of 19 episodes occurred. When compared to the O2 group, we noticed one hypoglycemic episode within 60 min from the OGTT start, 15 hypoglycemic episodes within 180 min from the OGTT, and two episodes within 240 min. In the control group, we did not observe any episodes of hypoglycemia.

### 5.4 Oxidative stress measurement

O1 and O2 patients O2 exhibited significantly higher concentrations of TOC and lower concentrations of TAC compared to control group (*p* < 0.05) (Table 3).

**TABLE 3:** Parameters of oxidative stress in the study group.

Parameter	TOC (umol/L)	O1/O2 vs. CG *p*-value**	O1 vs. O2 *p*-value **
**O1**	794.1 (173–2,298)	**0.018**	0.296
**O2**	821.5 (381–2,256)	**0.026**	
**CG**	598.7 (35.0–2,244)	-	**-**
**Parameter**	**TAC (umol/L)**	**O1/O2 vs. CG** *p*-value	**O1 vs. O2** *p*-value
**O1**	282 (216.5–374.4)	**0.022**	0.5873
**O2**	280.1 (180–391)	**0.048**	
**CG**	339.8 (227.2–662.9)	**-**	**-**

**TOC**, total oxidative capacity; **TAC**, total antioxidant capacity, **- Mann Whitney measurements; **O1**; overweight group.

**O2**, obese group; **CG**, control group.

**FIGURE 1:**
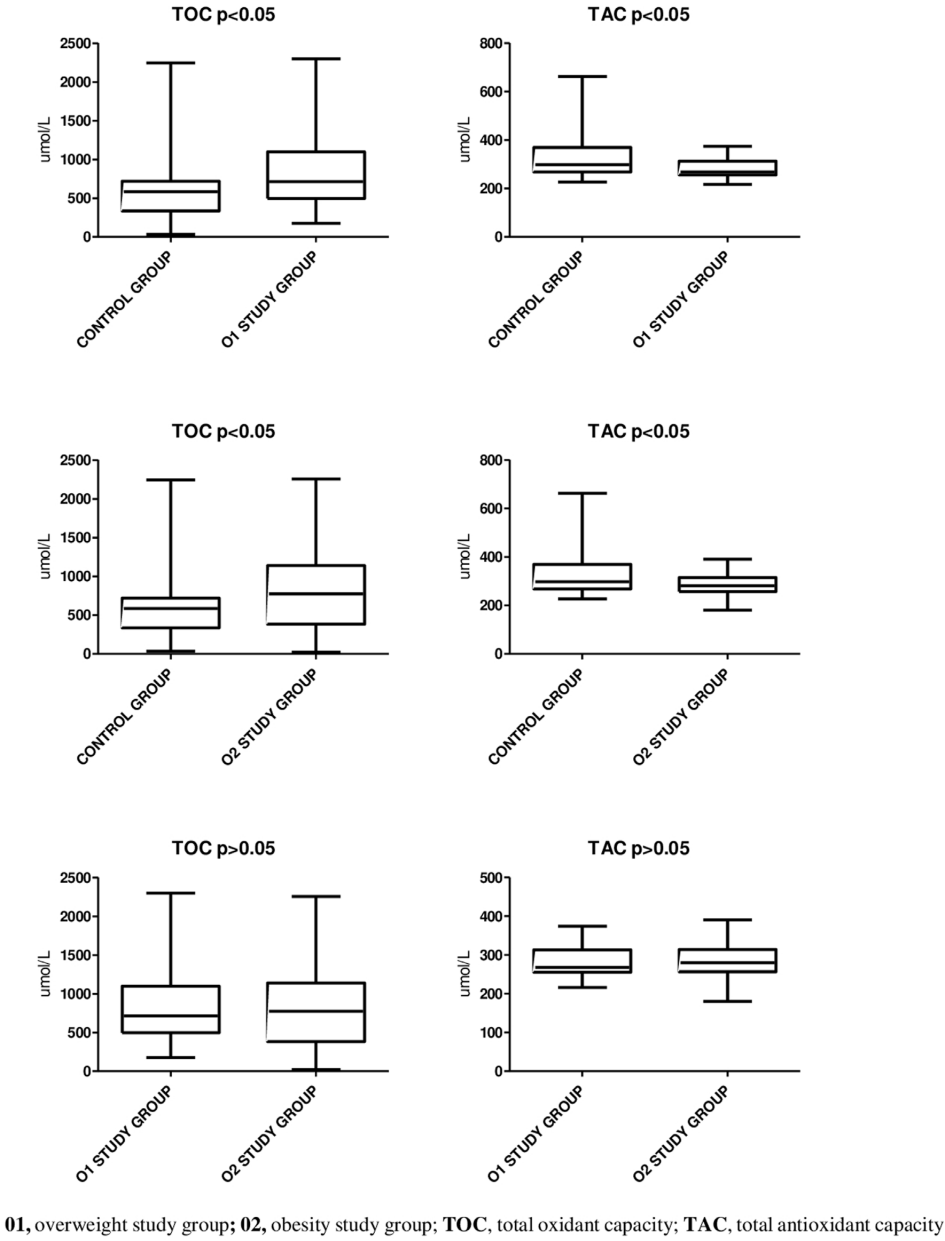
The oxidative status profiling.

However, there were no significant differences in oxidative status observed between O2 and O1 groups (*p* > 0.05) (Figure 1).

### 5.5 Oxidative stress measurement and reactive hypoglycemia

**FIGURE 2:**
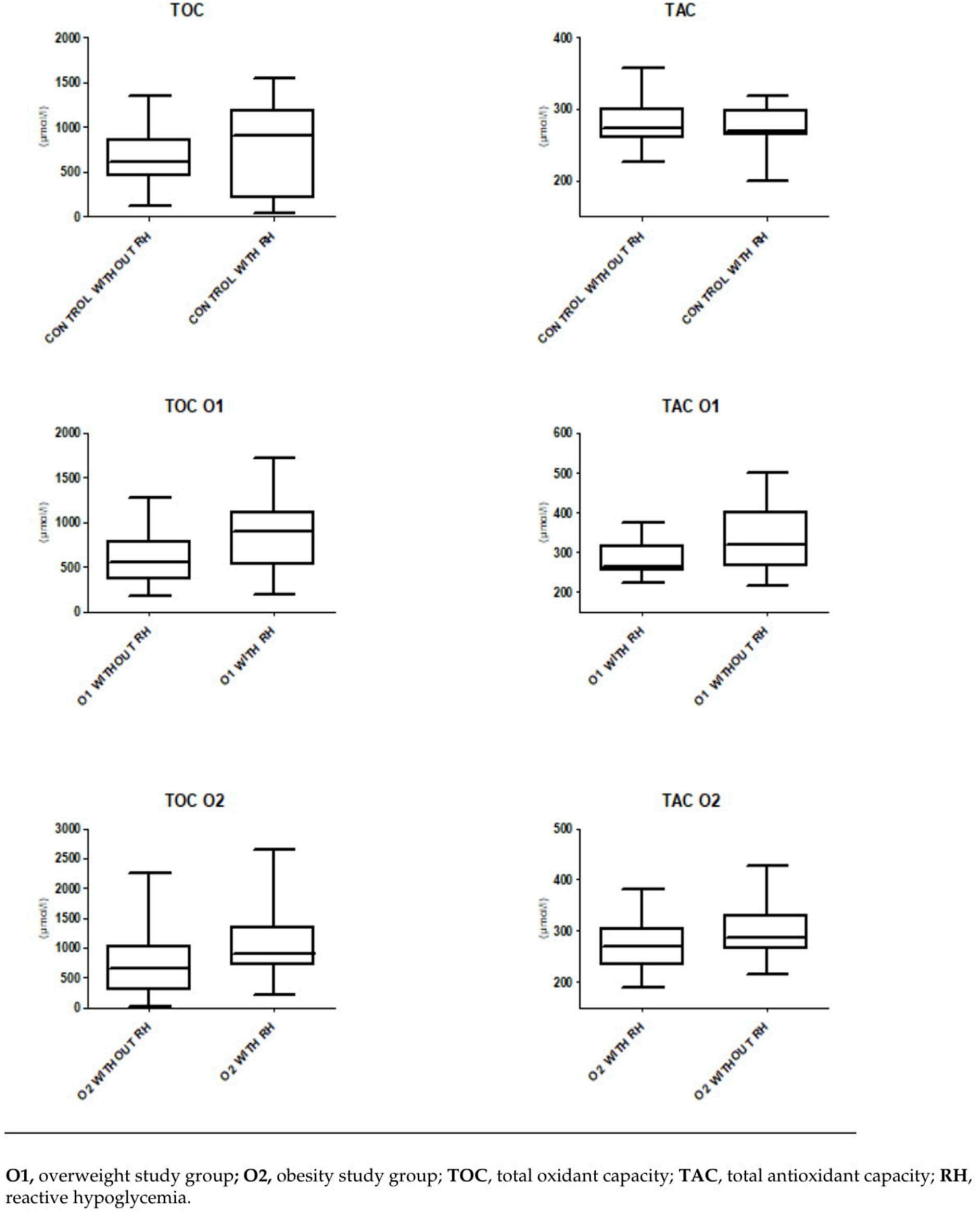
The oxidative status and reactive hypoglycemia.

Furthermore, higher concentrations of TOC and lower concentrations of TAC were observed in patients who experienced episodes of RH, both in the overweight and obese groups (Table 4; Figure 2). There were no statistically significant differences observed in the above-mentioned parameters concentrations in the control group with and without RH (Figure 2).

**TABLE 4:** Parameters of oxidative stress in the study group with and without reactive hypoglycemia.

Parameter	O1 with RH vs. O1 without RH *p*-value	O2 with RH vs. O2 without RH *p*-value **
**TOC (umol/L)**	**0.0165**	**0.0104**
	**O1 with RH vs. O1 without RH** *p*-value**	**O2 with RH vs. O2 without RH** *p*-value **
**TAC (umol/L)**	**0.0155**	**0.0262**

**TOC**, total oxidative capacity; **TAC**, total antioxidant capacity; **RH**, reactive hypoglycemia, **- Mann Whitney measurements.

### 5.6 Logistic regression analysis

To determine the origin of OS, whether they arise directly from the hypoglicemic episodes, logistic regression analysis was carried out. The results showed a noteworthy connection between hypoglycemic episodes and the levels of TOC/TAC among patients with increased body mass (all, *p* < 0.05) as presented in Table 5.

**TABLE 5:** Logistic regression analysis between hypoglycemic episodes and the levels of TOC and TAC.

Parameter	B	SE	p	OR (95% CI)
The oxidative status markers				
**TOC in O1 group**	−0.5876	0.0006681	**0.0421**	1.001 (0.9995–1.002)
**TOC in O2 group**	−1.492	0.0004253	**0.0274**	1.001 (1.000–1.002)
**TAC in O1 group**	3.223	0.004709	**0.0172**	0.9899 (0.9797–0.9983)
**TAC in O2 group**	2.359	0.005651	**0.0481**	0.9894 (0.9778–0.9999)

**TOC**, total oxidative capacity; **TAC**, total antioxidant capacity; **01,** overweight study group**; 02,** obesity study group; **B**, beta; **SE**, standard error; **p,** *p*-value; **OR,** odds ratio.

### 5.7 Correlations

Within the O1 study group, several notable correlations were observed. TOC exhibited a moderate positive correlation with body weight (R = 0.52; *p* < 0.02). Furthermore, TAC displayed a moderate negative correlation with body fat mass (R = −0.61; *p* < 0.01) and glucose concentration at 240 min during the OGTT (R = −0.48; *p* < 0.2). In the O2 group, similar correlations were identified. TOC demonstrated a moderate positive correlation with BMI (R = 0,25; *p* < 0.05), fat mass (R = 0.26; *p* < 0.05), and glucose levels at 240 min during the OGTT (R = 0.43; *p* < 0.04). Additionally, TOC exhibited negative correlations with muscle mass and TBW. Additionally, TAC exhibited negative correlations with body weight (R = −0.38; *p* < 0.05) and fasting glucose levels (R = −0.41; *p* < 0.05). In the analyzed O2 group, a moderate positive correlation was observed between TAC and TBW (R = 0.409). Additionally, a positive correlation between TAC and RMR (R = 0.411) was noted, along with a negative correlation with fat mass (R = −0.394). Furthermore, in the O1 group, a negative correlation between TOC and TAC was noted (R = −0.45, *p* = 0.001). No correlation between TOC and TAC was observed in the control group (Table 6). We observed a positive correlation between HOMA-IR and muscle mass (R = 0.35, R = 0.28), fat mass (R = 0.23, R = 0.33), WHR (R = 0.32, R = 0.18), and RMR (R = 0.30, R = 0.30) in both groups O1 and O2 (Table 7).

**TABLE 6:** Spearman’s correlation coefficients between parameters of OS status, body composition, WHR, and RMR in the O2 gorup.

O2 group	TOC		TAC	
	*p*-value	R	*p*-value	R
BMI (kg/m2)	0.019	0.25	-	
Fat mass (kg)	0.014	0.26	**<0.001**	−0.394
Muscle mass (kg)	0.003	−0.31	**-**	0.409
TBW	0.016	−0.25	**<0.001**	0.409
WHR	-	0.14	**-**	
RMR	0.028	−0.23	**<0.001**	0.411

**TOC**, total oxidative capacity; **TAC**, total antioxidant capacity; **BMI,** body mass index; **TBW**, total body water; **WHR**, waist to hips ratio; **RMR**, resting metabolic rate.

**TABLE 7:** Spearman’s correlation coefficients between HOMA-IR, Body Composition, WHR, and RMR in the O1 and O2 groups.

	O1 group HOMA-IRR	O2 group HOMA-IRR	*p*-value
BMI (kg/m2)	0.37	0.40	
Muscle mass (kg)	0.35	0.28	
Fat mass (kg)	0.23	0.33	**<0.05**
TBW	0.33	0.28	
WHR	0.32	0.18	
RMR	0.30	0.30	

**HOMA-IR,** insulin resistance; **BMI,** body mass index; **TBW**, total body water; **WHR**, waist to hips ratio; **RMR**, resting metabolic rate.

### 5.8 Diagnostic screening potential of the assessed parameters

**FIGURE 3:**
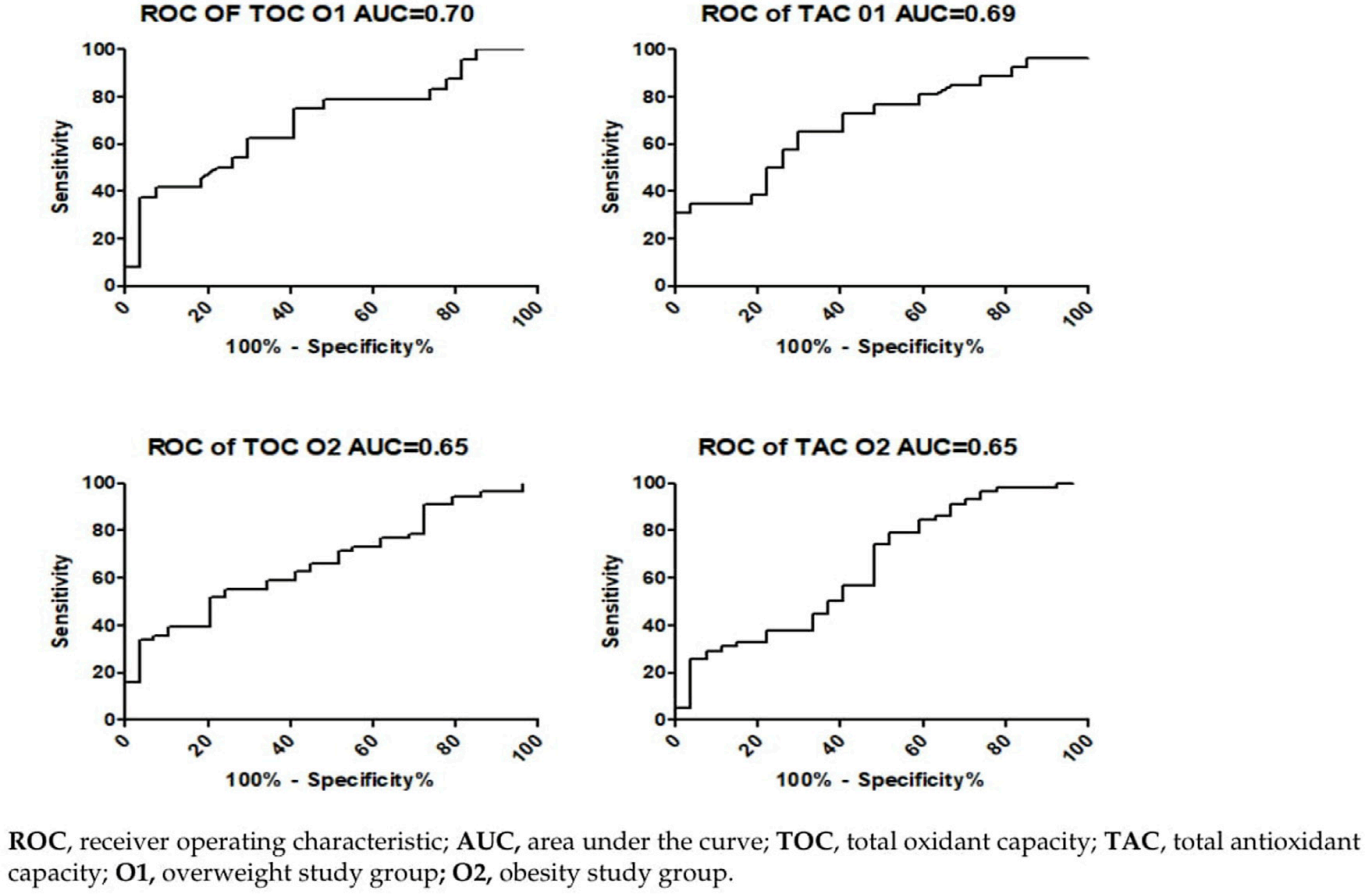
Evaluation of the specificity and sensitivity of TAC and TOC values as markers or RH based on the ROC curve.

To assess the diagnostic utility of the tested parameters, we calculated the ROC curve and presented an illustration depicting the relationship between sensitivity and specificity parameters in the ROC graphs (Figure 3). Cutoff values were determined using Youden’s index. To evaluate the diagnostic usefulness of serum concentrations of TAC and TOC as indicators of the risk of RH episodes, we calculated the areas under the ROC curves (AUCs) and compared them to AUC = 0.50 (the borderline of the diagnostic usefulness of a test). The highest AUC values were observed for plasma TOC concentrations and TAC concentrations in the O1 group (0.70 and 0.69, respectively). The TOC assay in the O2 group was characterized by an AUC of 0.65. Furthermore, the AUC value for TAC concentration in the same group of patients was calculated to be AUC = 0.65.

## 6 Discussion

Auth OS plays a crucial role in the development of obesity and its related complications. OS occurs when the body produces more ROS, such as free radicals, than it can neutralize with its antioxidant system (Slimen et al., 2014). Obesity is often associated with a low-grade chronic inflammatory state in adipose tissue, characterized by elevated levels of inflammatory markers and cytokines (Fernández-Sánchez et al., 2011; Kawai et al., 2021). As a result of this inflammation, fat cells release ROS, which further intensify the inflammatory state. This interplay between ROS and inflammation leads to increased OS (Vincent et al., 2007; Sankhla et al., 2012; Li and Yang, 2018). In obese individuals, there is an increased production of ROS in adipose tissues due to metabolic overload and excess fat (Liu et al., 2012). These ROS can cause damage to cells and tissues (Mancini et al., 2008). These processes contribute to the overproduction of adipokines and inflammatory mediators, which are significantly linked to the development of obesity and metabolic complications (Tafani et al., 2016; Godfrey et al., 2017; Nameni et al., 2017; Vecchié et al., 2018; Hörbelt et al., 2019; Francini et al., 2022). Additionally, ROS promote adipogenesis, lipogenesis, and the transformation of preadipocytes into mature fat cells, collectively contributing to the development of IR (de Ferranti and Mozaffarian, 2008; Tafani et al., 2016; Dludla et al., 2018; Mastrototaro and Roden, 2021). ROS also play a significant role in regulating appetite in hypothalamic neurons, which control energy balance (Liu et al., 2012; de Mello et al., 2018; Tsang et al., 2020). OS in obesity negatively impacts health by contributing to the development of IR, T2DM, vascular damage, increased risk of cardiovascular diseases, and accelerating the aging process (Petrie et al., 2018; Halim and Halim, 2019).

The main goal of our study was to conduct a comprehensive analysis, encompassing hormonal, oxidative, and antioxidant factors, to determine the potential role of OS in triggering or exacerbating IR in overweight and obese individuals. The results of our study unequivocally highlighted significantly intensified oxidative processes, as evidenced by increased levels of TOC, in overweight and obese individuals compared to the control group (*p* < 0.05). Higher OS indicators have also been observed in obese individuals in studies by other researchers (Rowicka et al., 2017; Xu et al., 2022). Simultaneously, obesity can lead to a decreased activity of the antioxidant system, meaning that the body struggles to neutralize ROS (Pérez-Torres et al., 2017; Zielińska-Bliźniewska et al., 2019). This may result from reduced antioxidant levels or decreased antioxidant effectiveness. Our results show that individuals with abnormal body weight had lower TAC levels (*p* < 0.05), confirming reduced antioxidant defenses. Other studies also confirm that obesity is characterized by persistent OS and reduced antioxidant levels in overweight and obese individuals (Vincent et al., 2007; Demircan et al., 2008; Pérez-Torres et al., 2017). These observations suggest that individuals with excess body weight are more susceptible to increased OS, which may significantly contribute to the development and progression of IR in this group. Importantly, the differences in oxidative status between the obese and overweight groups were not significantly different. Thus, this increased oxidative state in both groups appears to be closely related to the pathophysiology of obesity.

In the examined group of overweight individuals, we found significant associations between body mass and TOC concentration (R = 0.52; *p* < 0.02), as well as a negative correlation between body fat mass and TAC (R = −0.61; *p* < 0.01). In the overweight group, higher TOC levels correlated with body mass, consistent with the findings of other studies (Woo et al., 2012; Rowicka et al., 2017). Our study revealed significant negative correlations between TAC levels and body mass, in line with the results of Skalicky and others, who demonstrated increased OS in obese adults characterized by high levels of free radicals and reduced TAC (Skalicky et al., 2008). Simultaneously, we observed a negative correlation between TAC and glucose levels at 240′ minutes (R = −0.48) during the OGGT. Similar correlations were identified in the obese group. TAC negatively correlated with fasting glucose levels (R = −0.41; *p* < 0.05), suggesting a direct relationship between carbohydrate disturbances and OS and antioxidant depletion. Reduced antioxidant activity in individuals with carbohydrate disturbances and obesity may result from significant ROS production, depleting antioxidant enzymes and reducing the body’s antioxidant capacity. This may potentially explain the lower TAC levels observed in obese individuals.

It is worth noting that according to the literature, over 80% of obese individuals develop IR (Efremov et al., 2020). To explain the exacerbation of OS in obesity, we conducted insulin concentration studies during the OGTT, extending it to 240′ minutes. Insulin, a key hormone regulating glucose and lipid levels, was significantly elevated in obese individuals in our study, both in the fasting state and at 60′, 120′, 180′, and 240’ minutes after the glucose load OGTT (*p* < 0.001), consistent with previous reports (Hardy et al., 2012; Mirr et al., 2021). All obese patients in our study exhibited features of IR (HOMA-IR>2.5). Higher insulin and HOMA-IR levels were also observed in overweight individuals, in line with the results of previous studies (*p* < 0.001) (Martyn et al., 2008; Ferreira et al., 2009).

The everyday clinical practice highlights the significant issue of reactive hypoglycemia occurring in overweight and obese individuals. During our study, we observed that in the O1 group, there were 19 episodes of hypoglycemia within 180′ minutes from the start of the OGTT. When compared to the O2 group, we noticed 15 hypoglycemic episodes within 180′ minutes from the OGTT, and two episodes within 240′ minutes. Currently, several studies have demonstrated an association between hypoglycemia and OS, especially with regard to ROS production (Razavi et al., 2009; Papachristoforou et al., 2022). In this study, we demonstrate that individuals in the overweight development stage, who have encountered RH and obese individuals with a history of hypoglycemic episodes assessed through a prolonged OGTT test, exhibit notable variances in the examined OS parameters (*p* = 0.0165, *p* = 0.0104, respectively) and antioxidant capacity biomarkers compared to individuals with overweight or obesity without hypoglycemic episodes (*p* = 0.0155, *p* = 0.0262, respectively). Earlier investigations into hypoglycemia have shown that maintaining blood glucose levels (45 and 52.2 mg/dL), coupled with an extended duration of hypoglycemia (120′ vs 60′ min), resulted in a more notable elevation of pro-inflammatory mediators (Razavi et al., 2009; Gogitidze et al., 2010; Wright et al., 2010). Additionally, hypoglycemia induced by insulin in male subjects without diabetes was linked to heightened levels of proinflammatory cytokines, markers of lipid peroxidation, and increased production of ROS (Swinburn et al., 1991). The inflammation generated during hypoglycemia, in turn, can precipitate OS, further perpetuating the negative effects. The primary cause of tardive hypoglycemia has been largely linked to the heightened initial phase of insulin secretion as a result of IR. Moreover in the study we shown the diagnostic usefulness of serum concentrations of TAC and TOC as indicators of the risk of RH episodes. The highest AUC values were observed for plasma TOC and TAC concentrations in the O1 group (0.70 and 0.69, respectively). The TOC assay in the O2 group was characterized by an AUC of 0.65. Furthermore, the AUC value for TAC concentration in the same group of patients was calculated to be AUC = 0.65. The obtained results suggest that the future preventive utilization of OS marker concentration measurements in individuals with overweight and obesity, may enable the assessment of the risk of hypoglycemic episodes in these patients. The above results suggest that postprandial hyperinsulinemia followed by RH and an increase in OS may be a cause of overweight, and subsequently, obesity, which in turn leads to an increase in IR. Moreover, the results of our observation suggest that postprandial hypoglycemia in overweight individuals as often overlooked disorder can lead to increased chronic caloric intake and, subsequently, obesity what is in line with some studies analyzing risk factors for the development of obesity (Swinburn et al., 1991; Adabimohazab et al., 2016). In connection with this, the diagnostic utility of serum concentrations of TAC and TOC, it may allow for preventive measures and potentially reduce the negative long-term consequences of OS. In a healthy body, insulin acts as an antioxidant, protecting cells from damage by ROS (Song et al., 2018). It influences the production of antioxidant enzymes and can help maintain the balance between ROS and antioxidants. In obesity, cells in the body stop responding adequately to insulin, leading to elevated insulin levels in the blood (Kolb et al., 2020). This can lead to increased OS because excess insulin can activate signaling pathways that increase ROS production. Increased OS can contribute to the development of IR (Yaribeygi et al., 2019). Oxidative damage to cells and tissues can disrupt insulin signaling pathways, reducing the body’s ability to respond to this hormone. The increased presence of ROS in overweight and obese individuals may affect the apoptosis of Langerhans cells in the pancreas, disrupting the glucose-insulin balance and leading to hyperglycemia, further increasing OS levels (Robertson and Harmon, 2006). These processes contribute to the development of IR, a well-known consequence of obesity (Huang and Yang, 2016). Impaired insulin sensitivity, characteristic of IR, can explain the elevated levels of insulin and glucose in obese individuals.

In summary, insulin and OS have complex interrelationships. Insulin is a key hormone regulating blood glucose levels, but its excess or action under conditions of IR can lead to increased OS. Understanding these connections is essential for a better understanding of the mechanisms of development and treatment of metabolic diseases. Our results suggest potential interactions between OS parameters, body composition, and glucose metabolism dynamics in individuals with abnormal body weight. Overweight and, in later stages, obesity can lead to increased OS, potentially causing damage to cells and tissues. This can affect the body’s ability to counteract free radicals, which can be observed in changes in antioxidant capacity. The positive correlation with HOMA-IR suggests that individuals with a lower RMR may be more prone to IR (Maciak et al., 2020). A lower RMR may contribute to difficulties in maintaining a healthy body weight, which can, in turn, impact insulin sensitivity. Regarding glucose metabolism, overweight status can disrupt this pathway, inducing IR and affecting blood glucose levels. These disturbances can contribute to increased OS and potentially weaken the body’s ability to compensate for oxidative reactions (Mancini et al., 2007; Codoñer-Franch et al., 2011). Adipocytes secrete many hormones and cytokines which can affect energy homeostasis and the sensitivity of tissues to insulin and consequently have an impact on the glucose concentration. The primary reservoir of triglycerides in the body resides within adipose tissue, where they are released as nonesterified fatty acids and glycerol and then transport to other tissues. Muscle tissue can utilize these nonesterified fatty acids from adipose tissue, thereby providing a regulatory mechanism through which insulin can modulate the pace and outcome of glucose metabolism within muscle cells. Individuals with excess body weight often have a higher percentage of body fat relative to lean body mass, which influences insulin levels and consequently carbohydrate metabolism (Kershaw and Flier, 2004). Likewise, over the past 2 decades, research has revealed the capacity of skeletal muscle to function as an endocrine gland, emitting molecules known as “myokines.” Skeletal muscle can be susceptible to inflammation induced by obesity. Inflammation and IR triggered by obesity can also lead to the secretion of specific cytokine hormones from fat tissue and muscle called “adipo-myokines” (Merz and Thurmond, 2020). Skeletal muscle has the ability to release some kind of interleukin like IL-6, IL-8 and IL-15, along with irisin, myonectin, and myostatin (Raschke et al., 2013). The secretion of these circulating substances can either exacerbate or alleviate conditions related to obesity, inflammation, and IR. Additionally, skeletal muscle plays a key role in glucose disposal, and an increase in muscle mass has the potential to enhance the body’s efficiency in utilizing glucose, potentially reducing IR (Liu and Liu, 2019). However, previous studies in mice have shown that increasing IR in obesity is associated with a receptor-level defect and internal changes in the insulin signaling pathway, without a significant alteration in the number of the GLUT-4 glucose transporter responsible for insulin sensitivity (Le Marchand-Brustel et al., 1999). What is important this defect appears very early in the development of obesity. In our study we noted positive correlation between muscle mass and HOMA implies that individuals with greater skeletal muscle mass might experience enhanced IR due to a potential defect in the functioning of the GLUT-4 receptor. A positive correlation with HOMA indicates that individuals with a higher WHR, reflecting a greater concentration of abdominal fat, might face an elevated risk of IR. In the context of obesity, which is associated with a state of chronic, mild, low-grade inflammation and observed negative impact on GLUT-4 function, the body requires higher levels of insulin to facilitate glucose uptake into cells, including muscle cells. Furthermore, obese patients are characterized by a downregulation of GLUT genes (Deal et al., 2018). In obese individuals, increasing muscle mass does not necessarily lead to a reduction in IR in the context of the mentioned disorders. This is a significant factor in the development of T2DM, a condition closely linked to obesity and IR. To elucidate the role of OS in obesity, we conducted body composition analyses using bioimpedance methods. The observed adverse correlation between fat content and TAC in obese individuals is likely associated with the fact that adipose tissue is metabolically active and can produce inflammatory molecules and oxidative byproducts (Ibrahim, 2010; Unamuno et al., 2018). This, in turn, may contribute to reduced antioxidant capacity within the body. The positive correlation with HOMA underscores the connection between higher levels of adipose tissue and IR. Excess adipose tissue, particularly in the abdominal region, is recognized for its role in contributing to IR, and this correlation reinforces the impact of adiposity on impaired insulin sensitivity. Assessing OS status in this context may have the potential as a diagnostic tool for identifying individuals at increased risk of developing IR and associated complications. The positive correlation with HOMA suggests that individuals with a lower RMR may be more susceptible to IR. A lower RMR can contribute to challenges in maintaining a healthy body weight, which, in turn, can affect insulin sensitivity.

To sum it up, these positive correlations with HOMA highlight the significance of these factors in the context of IR. Lower fat tissue mass, especially in the abdominal area, are linked to improved insulin sensitivity, whereas a higher WHR and lower RMR may be associated with an elevated risk of increased OS status and in consequence IR development (Sepandar et al., 2019; Haines et al., 2020). These findings have implications for assessing the risk of T2DM and emphasize the importance of lifestyle changes, such as dietary control and physical activity, to address and alleviate IR in clinical practice.

However, it is important to note the limitations of OS indicators such as TAC and TOC when interpreting results. These indicators have a short half-life and do not always exclusively indicate OS. They can also be influenced by other variables, potentially leading to misinterpretation of results (Dhama et al., 2019; Puttabyatappa et al., 2020). Levels of OS indicators can vary significantly among individuals due to genetic, environmental, and lifestyle factors, making it challenging to establish universal reference ranges. Therefore, the results of TAC and TOC studies should be carefully considered in a specific clinical context. Nevertheless, the clinical significance of measuring TAC and TOC status can enhance our understanding of the clinical course of overweight and obesity. It may also provide a means of monitoring responses to therapeutic interventions.

## 7 Conclusion

The findings of this study provide valuable insights into the intricate relationship between OS and IR in overweight and obese individuals. Our results emphasize the importance of evaluating OS in this population, as it seems to be closely connected to glucose metabolism and body composition. Our findings may suggest that individuals with overweight or obesity could be at a heightened risk of IR compared to those with a normal body weight. This observation holds clinical significance concerning the assessment of T2DM risk and the necessity for proactive preventive measures. Moreover, in the study, we demonstrated the diagnostic utility of serum concentrations of TAC and TOC as indicators of the risk of RH, the occurrence of which, even at the stage of overweight, may be associated with increased OS and further development of obesity. However, it is important to recognize that further research is necessary to delve deeper into the mechanisms by which increased OS contributes to the development of IR. Early detection of obesity and timely initiation of therapeutic interventions are crucial strategies for preventing the harmful effects of free radicals and the associated multi-organ complications. The results of our observation, which suggest increased OS and the resulting rise in IR in overweight and obese patients, indicate the potential for implementing interventions aimed at impro ving insulin sensitivity and antioxidant capacity through dietary changes, physical activity, or antioxidant supplements. This could lead to enhanced therapeutic outcomes for these patients. This proactive approach has the potential to pave the way for more effective strategies in managing and preventing metabolic disorders associated with obesity. Furthermore, integrating serum OS marker measurements into routine clinical practice may open the door to more personalized and precise therapeutic approaches for individuals with overweight, obesity, and concurrent IR.

## Data Availability

The original contributions presented in the study are included in the article/Supplementary Material, further inquiries can be directed to the corresponding authors.
